# Father Phubbing and Adolescents’ Depressive Symptoms: The Roles of Relationship Satisfaction and Need to Belong

**DOI:** 10.3390/bs15020139

**Published:** 2025-01-27

**Authors:** Jilong Wang, Pengcheng Wang, Yongjie Yue, Lipeng Yin, Wei Wang, Guohua Zhang, Yulong Yin

**Affiliations:** 1 School of Media and Communication, Shanghai Jiao Tong University, Dongchuan Road 800, Shanghai 200240, China; wangjilong88@163.com (J.W.); tianhai001@126.com (P.W.); 2 School of Journalism and Communication, Tsinghua University, Beijing 100084, China; yueyj2020@163.com; 3 Department of Psychology, The University of Hong Kong, Hong Kong; 4 Department of Psychology, Datong University, No. 405 Xingyun Street, District, Datong 037009, China; wangwei05028@126.com; 5 Department of Psychology, Wenzhou Medical University, University Town, Chashan, Wenzhou 325035, China; zghcnu@wmu.edu.cn; 6 School of Psychology, Northwest Normal University, Lanzhou 730070, China; yinhaomin2486144@163.com

**Keywords:** father phubbing, depressive symptoms, relationship satisfaction, need to belong, adolescents

## Abstract

Recent research indicates that parental phubbing is a risk factor for adolescents’ depressive symptoms; however, limited research has examined the association between father phubbing and adolescents’ depressive symptoms. The present study tested the association between father phubbing and adolescents’ depressive symptoms. Furthermore, the mediating and moderating effects underlying this link were examined. A total of 1319 participants (39.5% were boys, mean age = 16.15 years) completed measures regarding father phubbing, father–child relationship satisfaction, depressive symptoms, and the need to belong. By using a two-wave longitudinal design, the results showed that father–child relationship satisfaction mediated the association between father phubbing and adolescents’ depressive symptoms, and the need to belong exacerbated the association between father phubbing and father–child relationship satisfaction. Limitations and implications of this study were elaborated.

## 1. Introduction

Phubbing, as a common social phenomenon in this digital era, refers to the act of subbing other people in social settings due to own mobile phone use (Haigh, 2015; Yin et al., 2024a). It is closely related to, but distinct from, the concept of “technoference”, which refers to everyday interruptions in interpersonal interactions or shared time caused by digital and mobile technology (McDaniel, 2015). Technoference can occur during face-to-face conversations, routines such as mealtimes or play, or through the perceived intrusion when one person uses digital devices during shared moments (McDaniel & Radesky, 2018). A recent study showed that 89% of people reported that they exhibited phubbing behavior (Ranie & Zickuhr, 2015). Phubbing also takes place frequently in China due to the fact that about 74% of Chinese people owned a mobile phone by June, 2022 (China Internet Network Information Center, 2022). Given the wide popularity of phubbing, increasing academic attention has been paid to the social outcomes caused by phubbing in recent years (Bai et al., 2020a; David & Roberts, 2021; P. Wang et al., 2022b; X. Wang et al., 2020b; P. Wang et al., 2025; Yasin et al., 2020; Yin et al., 2024a, 2024b). Particularly, a large proportion of the literature has focused on uncovering the potential influences of parental phubbing on adolescent mental health (Niu et al., 2020; Tong et al., 2023; X. Wang et al., 2020a, 2020b; Xie & Xie, 2020). Although a few studies have indicated that parental phubbing is positively related to adolescents’ depressive symptoms (Bai et al., 2020b; X. Wang et al., 2020a; Xie & Xie, 2020), no existing research has examined the association between father phubbing and adolescents’ depressive symptoms by longitudinal data. Further, the internal mechanisms underlying this association also remain largely unexplored.

Depressive symptoms have been identified as one of the most destructive factors for adolescent development; for instance, depressive symptoms are closed related to anxiety (Jacobson & Newman, 2017), loneliness (Peerenboom et al., 2015), problematic internet use (Gao et al., 2021), and even suicidal thoughts and behaviors (Franklin et al., 2017). Understanding the factors that contribute to adolescent depressive symptoms is crucial, particularly in the context of rapid technological changes. As the neo-ecological theory posits that development occurs within an interplay of physical and virtual microsystems (Navarro & Tudge, 2023), the influence of technology-mediated interactions, such as phubbing, on adolescents’ psychological well-being cannot be overlooked. These interactions often disrupt proximal processes within critical relationships, such as those with parents, potentially amplifying adverse developmental outcomes, including depressive symptoms. Given the adverse influences of depressive symptoms and popularity of phubbing in modern society, examining the internal mechanisms in the relationship between father phubbing and adolescents’ depressive symptoms would be beneficial to inspire prevention and intervention targeted at reducing adolescents’ depressive symptoms. Therefore, the present study aimed to extend the existing literature by uncovering the mediating effect of relationship satisfaction and the moderating effect of the need to belong in this association by using two-wave longitudinal data.

### 1.1. The Mediating Role of Relationship Satisfaction

Although not yet empirically examined, it is possible that father phubbing can negatively predict father–child relationship satisfaction. Based on prior research, father phubbing indicates a scene where a father is using his phone instead of interacting with his child (Geng et al., 2021; P. Wang et al., 2022a). As the displacement theory posits (Kraut et al., 1998), people’s new media use can occupy the time that could have been spent on meaningful social interactions with people in real life. Based on this theory, father phubbing may impair father–child communication and make the child feel less cared for by his/her father, which can damage the child’s perceived relationship satisfaction with his/her father. Although no research has directly examined this assumption, existing empirical evidence roughly supports this notion. For instance, a few studies have found that partner phubbing is negatively related to relationship satisfaction among couples (Roberts & David, 2016; X. Wang et al., 2017). Furthermore, several studies have indicated that parent phubbing can damage the parent–child bond, including mother–child communication (P. Wang et al., 2021), perceived mother acceptance (Qu et al., 2020), parent–child attachment (Xie et al., 2019), and the parent–child relationship (Hong et al., 2019; Liu et al., 2021). Based on the above literature, it is possible that father phubbing would negatively predict father–child relationship satisfaction. To date, no existing study has tested this association in the literature.

Sufficient research suggests that father–child relationship satisfaction can predict adolescents’ depressive symptoms. According to the ecological system theory (Bronfenbrenner, 1977), family is a vital part of the microsystem in a person’s life, which can directly influence a person’s developmental outcomes. Based on this theory (Bronfenbrenner, 1977), the parent–child relationship, as an important element in the family setting, can significantly impact people’s psychological status, such as depressive symptoms. Empirical studies are in line with this notion. For instance, cross-sectional studies show that the parent–child relationship is negatively associated with people’s depressive symptoms (Bradford et al., 2017; Guo et al., 2015; Pina-Watson & Castillo, 2015). Furthermore, longitudinal research also suggests that the parent–child relationship can predict people’s depressive symptoms (Branje et al., 2010). Thus, it is convincing that father–child relationship satisfaction can predict adolescents’ depressive symptoms.

Summing up, it is possible that father phubbing can negatively predict father–child relationship satisfaction, which in turn can negatively predict adolescents’ depressive symptoms. That is, father–child relationship satisfaction may mediate the association between father phubbing and adolescents’ depressive symptoms. To date, no empirical research has examined this mediating effect. Based on the above literature, we establish the first hypothesis in our study:

**Hypothesis** **1.** *Father–child relationship satisfaction would mediate the association between father phubbing and adolescents’ depressive symptoms.*

### 1.2. The Moderating Role of the Need to Belong

Although father phubbing may predict father–child relationship satisfaction, this effect can be different due to adolescents’ diverse personality characteristics. Thus, it would be beneficial to examine potential moderating roles that can impact the effect of father phubbing on father–child relationship satisfaction. This study tested an assumption that the need to belong would moderate the relationship between father phubbing and father–child relationship satisfaction.

As the differential susceptibility hypothesis suggests (Belsky & Pluess, 2009), people with different personal attributes can diversely respond to the same environmental stimulates. The need to belong is a fundamental, powerful, and pervasive human motivation that has strong and multiple influences on people’s cognitive and emotional patterns (Baumeister & Leary, 1995). In addition, the self-determination theory (SDT) emphasizes that relatedness, the sense of being connected to others, is a basic psychological need essential for people’s well-being (Deci & Ryan, 2012). People whose need to belong is high are more sensitive about their social bonds with other people and they often pay more attention to their social interactions with others (Baumeister & Leary, 1995; Leary et al., 2013). Thus, they are more likely to be impacted by negative interpersonal interactions, such as phubbing behavior. Given that parental phubbing sends a clear message to adolescents that their parents view mobile phones to be more important than them, this often makes adolescents feel ignored or rejected by their parents (Stockdale et al., 2018; X. Wang et al., 2020a). It is theoretically possible that father phubbing can cause more damage to father–child relationship satisfaction among adolescents with a higher level of need to belong. Empirical evidence indirectly supports this notion. For instance, existing studies show that the need to belong moderates the association between interpersonal relationships and people’s behaviors related to relationship seeking (Hamilton & Dehart, 2019; P. Wang et al., 2017). To our knowledge, no research has tested the moderating role of the need to belong in the link between father phubbing and father–child relationship satisfaction. Based on the literature discussed, we come up with the following assumption:

**Hypothesis** **2.** *The need to belong would moderate the association between father phubbing and father–child relationship satisfaction*.

## 2. The Current Study

**Figure 1:**
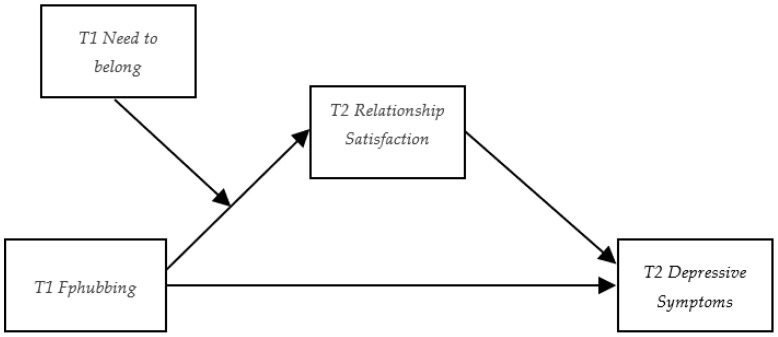
The proposed moderated mediation model. Note: Fphubbing = father phubbing.

In the current study, we used a longitudinal study design to uncover how father phubbing can predict adolescents’ depressive symptoms (Figure 1). In particular, the aims of the present study include the following: (a) to examine whether father–child relationship satisfaction would mediate the link between father phubbing and adolescents’ depressive symptoms and (b) to test whether the need to belong would moderate the link between father phubbing and father–child relationship satisfaction. Taken together, the integrated research model can address queries regarding both mediating (i.e., how does father phubbing predict adolescents’ depressive symptoms) and moderating (i.e., for whom is the impact of father phubbing on father–child relationship satisfaction more severe) effects in a single model.

## 3. Method

### 3.1. Participants

Participants included adolescents who took part in the parental phubbing on adolescent development project, which was a longitudinal study aimed at exploring the influences of parental phubbing on adolescent development. Two waves of data were examined in this study. In particular, the data of Time 1 and Time 2 were collected during the summer of 2019 and the winter of 2019, respectively. We recruited the participants from two senior high schools by convenience sampling in Jiangsu and Hebei Provinces in China. The survey was approved by the corresponding research ethics committee. Before data collection, informed consent was acquired from the participants and their teachers. The questionnaires were filled out by the participants in their classrooms. The participants were informed that they were free to leave the study if they desired. The required sample size was predetermined using G*Power software (version 3.1.9.7), based on a medium effect size (d = 0.30), α = 0.05, and power = 0.99, suggesting that at least 188 participants were required for the study. At Time 1, 1352 adolescents completed measures on demographic information, father phubbing scale, and the need to belong. At Time 2, 1319 adolescents who completed the first assessments completed the measures on father–adolescent relationship satisfaction and depressive symptoms. We included participants who lived with their fathers during the study period, provided complete data for all measures at both Time 1 and Time 2, and passed the attention test. A total of 1319 valid responses were retained for the final analysis. The attrition analyses showed that participants dropping out of the survey after Time 2 were not statistically different to the measurements at Time 1 from those who participated in the survey for both times. Regarding their demographic information, the mean age of the participants was 16.15 years (*SD* = 0.65), ranging from 10 to 19 years old. Furthermore, 39.5% of the participants were boys, 77.5% of them lived in the rural areas, and 17.8% of them were the only child in their family. Moreover, 35.8% of the participants’ fathers and 32.5% of the participants’ mothers had a high school degree or above.

### 3.2. Ethical Considerations

This study was approved by the institutional review board (IRB) of the author’s university. Informed consent was obtained from the participants. The participants were informed that they could leave the study anytime they wanted. The data obtained in this study were anonymized. The participants received a small gift for their participations (i.e., a pen).

### 3.3. Measures

#### 3.3.1. Father’s Phubbing

The 22-item father phubbing scale, which was modified from the Generic Scale of Being Phubbed (Chotpitayasunondh & Douglas, 2018), was applied to assess perceived father phubbing. A representative item is “My father would rather pay attention to his phone than talk to me”. Participants rated each item on a 7-point Likert scale ranging from 1 (*completely not true*) to 7 (*completely true*). Responses to these items were averaged, with higher average scores representing higher levels of perceived father phubbing. Cronbach’s α was 0.91 for the current sample.

#### 3.3.2. Depressive Symptoms

The 20-item Center for Epidemiological Studies Depression Scale (Radloff, 1977), which has been widely used among Chinese participants (P. Wang et al., 2018), was applied to assess adolescents’ depressive symptoms. A representative item is “I felt depressed”. Participants rated each item on a 4-point Likert scale ranging from 1 (*rarely or none of the time*) to 4 (*most or all of the time*) to indicate experienced depressive symptoms during the last week. Responses to these items were averaged, with higher average scores representing higher levels of depressive symptoms. Cronbach’s α was 0.95 for the current sample.

#### 3.3.3. Father–Adolescent Relationship Satisfaction

The 4-item father–child relationship satisfaction scale, which was modified from the Relationship Satisfaction Scale (Murray et al., 2015), was applied to assess adolescents’ relationship satisfaction with their father. Participants rated each item on a 7-point Likert scale ranging from 1 (*not at all*) to 7 (*completely true*). Responses to these items were averaged, with higher average scores representing higher levels of father–adolescent relationship satisfaction. Cronbach’s α was 0.89 for the current sample.

#### 3.3.4. Need to Belong

The Single-Item Need to Belong Scale (Nichols & Webster, 2013), which has been widely used among Chinese participants (P. Wang et al., 2020), was applied to assess adolescents’ need to belong. The item is “I have a strong need to belong”. Participants rated this item on a 7-point Likert scale ranging from 1 (*not at all*) to 7 (*extremely*), with higher scores representing higher levels of need to belong.

### 3.4. Data Analysis

First, we excluded responses with missing data in the analyses process. Second, descriptive statistics and Pearson correlations among the variables of interest were calculated. Third, the PROCESS macro for SPSS (Model 4) was used to calculate the hypothesized mediating effect (Hayes, 2017). At last, the PROCESS macro for SPSS (Model 7) was used to calculate the hypothesized moderated mediation effect (Hayes, 2017). All variables were standardized before the analyses in the second and third step.

## 4. Results

### 4.1. Descriptive Analyses and Bivariate Analyses

The descriptive statistics and Pearson correlations for variables are shown in Table 1. As we can see, T1 father phubbing was negatively associated with T2 father–adolescent relationship satisfaction (*r* = −0.28 ***, *p* < 0.001) and positively associated with T2 adolescents’ depressive symptoms (*r* = 0.19 ***, *p* < 0.001). Furthermore, T2 father–adolescent relationship satisfaction was negatively associated with T2 adolescents’ depressive symptoms (*r* = −0.25 ***, *p* < 0.001). Moreover, T1 need to belong was positively associated with T1 father phubbing (*r* = 0.14 ***, *p* < 0.001), and negatively associated with T2 father–adolescent relationship satisfaction (*r* = −0.08 **, *p* < 0.001) and positively associated with T2 adolescents’ depressive symptoms (*r* = 0.18 ***, *p* < 0.001). Given that age and gender were not significantly related to any other variables, they were not included as covariates in the subsequent analyses.

### 4.2. Examining the Mediation Effect

We assumed that father–adolescent relationship satisfaction would be a mediator in the link between father phubbing and adolescents’ depressive symptoms, which was examined by the PROCESS macro (Model 4; Hayes, 2017). As shown in Table 2, T1 father phubbing negatively predicted T2 father–adolescent relationship satisfaction (*β* = −0.28, *p* < 0.001), and T2 father–adolescent relationship satisfaction was negatively associated with adolescents’ T2 depressive symptoms (*β* = −0.21, *p* < 0.001). In the meantime, the residual direct effect of T1 father phubbing and T2 adolescents’ depressive symptoms remained significant (*β* = 0.13, *p* < 0.001). The bootstrap analyses showed that the indirect effect in the model was 0.06, *SE* = 0.01, 95% CI [0.04, 0.08]. The mediation effect size can be classified as small to moderate (Hayes, 2017). Thus, T2 father–adolescent relationship satisfaction mediated the relationship between T1 father phubbing and T2 adolescents’ depressive symptoms.

### 4.3. Examining the Moderated Mediation Effect

**Figure 2:**
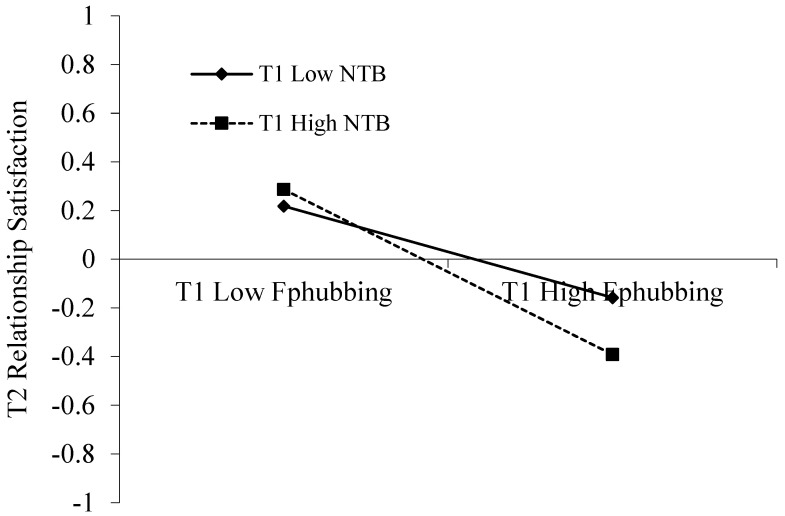
The moderating role of need to belong. Note: Fphubbing—father phubbing, NTB—need to belong.

We hypothesized that need to belong would moderate the association between father phubbing and father-adolescent relationship satisfaction, which was examined by the PROCESS macro (Model 7; Hayes, 2017). As shown in Table 3, the interaction of T1 father phubbing and T1 need to belong negatively predicted T2 father-adolescent relationship satisfaction (*β* = −0.08, *p* < 0.001). To make the results visual, we plotted T1 father phubbing on T2 father-adolescent relationship satisfaction, separately at a high and low levels of T1 need to belong (see Figure 2). Simples slope analyses indicated that the effect of T1 father phubbing on T2 father-adolescent relationship satisfaction was significantly stronger among adolescents with a high level of need to belong (*β_simple_* = −0.34, *p* < 0.001) than among adolescents with a low level of need to belong (*β_simple_* = −0.19, *p* < 0.001). Moreover, the index of moderated mediation was 0.02. *SE* = 0.007, Cis = [0.003, 0.030]. This value indicates a small to moderate effect size (Hayes, 2017). Thus, need to belong moderated the mediation effect of father phubbing on adolescents’ depressive symptoms via father-adolescent relationship satisfaction.

## 5. Discussion

Our study examined the underlying mechanisms between father phubbing and adolescents’ depressive symptoms by examining a moderated mediation model through a longitudinal study design. The findings indicated that father–child satisfaction mediated the association between father phubbing and adolescents’ depressive symptoms, and the need to belong exacerbated the association between father phubbing and father–child satisfaction.

### 5.1. The Mediating Role of Father–Child Satisfaction

Firstly, our study showed that father phubbing can predict adolescents’ depressive symptoms, which allies with previous studies showing that parent phubbing is positively associated with adolescents’ depressive symptoms (Bai et al., 2020b; P. Wang et al., 2022b; X. Wang et al., 2020a; Xie & Xie, 2020; Al-Saggaf, 2022). This finding suggests that father phubbing can be a risk factor for adolescent development. A possible explanation is that fathers’ involvement plays a crucial role in adolescents’ mental health outcomes (Li, 2020). Prior studies have indicated that the frequency of fathers’ contact with their children, and the child-rearing practices of the adults with whom children live, are strongly associated with children’s social and psychological adjustment (Xu & Yeung, 2013). Thus, father phubbing may restrict meaningful interactions with their children, contributing to adolescents’ depressive symptoms.

Secondly, our study showed that father–child satisfaction mediated the association between father phubbing and adolescents’ depressive symptoms. That is, father–child satisfaction can be one of the reasons why father phubbing can predict adolescents’ depressive symptoms. To our knowledge, our study is the first to examine this effect in the literature. To be specific, these findings show that early father phubbing can predict later adolescents’ depressive symptoms, which supports the displacement theory (Kraut et al., 1998). As the displacement theory assumes (Kraut et al., 1998), people’s media use can substitute for the time that could have been spent on more meaningful interactions with others in real life. Thus, it is reasonable to find that father phubbing, as a form of improper mobile phone use in the family context, can contribute to adolescents’ depressive symptoms. This finding further confirms the associations provided by prior research showing that parent phubbing is negatively related to the parent–child relationship (Błachnio, 2024; Mohammad Hussin et al., 2024; Liu et al., 2021). Moreover, it is important to consider the social, economic, and cultural context when interpreting these findings. In Chinese culture, where family harmony, respect, and interdependence are highly valued, father phubbing may lead to stronger feelings of rejection and conflict, as it disrupts these fundamental family ideals (Cheung et al., 2020; Lam et al., 2012). The role of the father in Chinese families, traditionally as a figure of authority and provider (Li, 2020), further exacerbates the negative impact of phubbing, as adolescents may perceive it as a failure to fulfill these familial expectations. In contrast, in Western cultures, where autonomy is emphasized, phubbing may be seen more as a sign of diminished emotional connection or lack of prioritization in relationships (Błachnio, 2024; Kildare & Middlemiss, 2017). Additionally, these findings also show that father–child relationship satisfaction is positively related to adolescents’ depressive symptoms, which allies with the ecological system theory (Bronfenbrenner, 1977). Based on this theory (Bronfenbrenner, 1977), the parent–child relationship is an important element in the microsystem, which would significantly impact adolescents’ developmental outcomes, including depressive symptoms. This finding adds empirical evidence to the notion that a negative parent–child relationship can lead to adolescents’ depressive symptoms (Branje et al., 2010; Chiang & Bai, 2022).

### 5.2. The Moderating Role of Need to Belong

Our study also assumed that the need to belong would moderate the association between father phubbing and father–child relationship satisfaction. The results reveal that the need to belong exacerbates this association. In particular, the effect between father phubbing and father–child relationship satisfaction is much stronger among adolescents with a higher level of need to belong compared with those with a lower level of need to belong, which is in line with the differential susceptibility hypothesis (Belsky & Pluess, 2009). According to the differential susceptibility hypothesis (Belsky & Pluess, 2009), people with diverse personal traits may respond differently to the same environmental factors. From a developmental standpoint, this effect may be particularly pronounced during adolescence, a critical period characterized by heightened sensitivity to social relationships and a strong desire for connection and belonging (Crone & Dahl, 2012; Rejaän et al., 2022). For adolescents with a high need to belong, they care more about their social relationships with other people compared with those with a low need to belong (Baumeister & Leary, 1995; Leary et al., 2013). During this developmental stage, the strong desire for meaningful connections makes adolescents particularly vulnerable to negative interpersonal interactions, such as father phubbing. When their need to belong is unmet, it can result in a deeper sense of rejection and dissatisfaction within the father–child relationship. Thus, the effect between father phubbing and relationship satisfaction is much stronger among adolescents with a higher need to belong.

### 5.3. Limitations and Implications

Some limitations should be noticed when interpreting the findings in our study. First, our study utilized a two-wave longitudinal design to examine the mediating effect. However, as variables were not measured in both waves, this limited our ability to determine the temporal order of father–child relationship satisfaction and adolescents’ depressive symptoms. It is possible that the relationships examined in the research model could be bidirectional. Thus, it would be beneficial for future research to use three-wave longitudinal data or an experimental method to further confirm the results provided in our study. Moreover, future research can track and analyze the developmental trajectory of the relationships between the above variables across age through long-term longitudinal studies. Second, our study used the self-report method to collect data about father phubbing, which may have limited the validity of the results. It would be better to use data from diverse sources (e.g., fathers and their children) to further confirm the results found in our study. Third, our study used a convenience sampling method, which only recruited participants from Chinese students, which limited the generalizability of the findings. Future research should verify findings from different populations. Fourth, the need to belong was measured with a single item, which might not fully capture this construct. Multi-item scales are recommended for future research. Fifth, the majority of participants were girls, which could limit the generalizability of the findings. Future studies should aim for a more gender-balanced sample to explore potential gender differences.

Despite the above-mentioned limitations, our study has some helpful implications. Firstly, our study illuminates that father–child relationship satisfaction mediates the association between father phubbing and adolescents’ depressive symptoms, and the need to belong exacerbates the association between father phubbing and father–child relationship satisfaction. All these findings can deepen our understanding of how father phubbing can contribute to adolescents’ depressive symptoms, and for whom the effect of father phubbing on father–child relationship satisfaction is more severe. Moreover, the findings in our study have meaningful practical implications. For instance, our study shows that father phubbing can predict adolescents’ depressive symptoms via father–child relationship satisfaction, which reminds fathers that they should reduce their phubbing behaviors in the family context. Furthermore, our study shows that the adverse effect of father phubbing on father–child relationship satisfaction is more severe among adolescents with a high need to belong, which can remind practitioners aiming to improve father–child relationships to pay more attention to these adolescents.

## 6. Conclusions

In sum, this study shows that early father phubbing can predict later adolescents’ depressive symptoms. Moreover, this relation is mediated by father–child relationship satisfaction. In addition, the relation between father phubbing and father–child relationship satisfaction is exacerbated by adolescents’ need to belong, with the effect being much stronger among adolescents with a higher level of need to belong. This study underscores that father phubbing can be one of these important factors for prevention and intervention in adolescents’ depressive symptoms. This study provides a sophisticated understanding of adolescents’ depressive symptoms in this digital era.

## Figures and Tables

**Table 1:** Descriptive statistics and correlations among variables of interest.

Variables	*M*	*SD*	1	2	3	4	5	6
1. T1 Father phubbing	2.25	0.86	1					
2. T2 Relationship satisfaction	5.10	1.48	−0.28 ***	1				
3. T2 Depressive symptoms	2.05	0.65	0.19 ***	−0.25 ***	1			
4. T1 Need to belong	3.86	1.87	0.14 ***	−0.08 **	0.18 ***	1		
5. Gender	0.61	0.49	0.02	−0.001	0.04	0.02	1	
6. Age	16.16	0.64	−0.02	0.00	−0.02	−0.02	−0.11 ***	1

Note: *N* = 1319. *M*—mean, *SD*—standard deviation. ** *p* < 0.01. *** *p* < 0.001. Gender: male = 0, female = 1.

**Table 2:** Testing the mediation effect of relationship satisfaction.

	Model 1 (T2 RS)				Model 2 (T2 Depressive Symptoms)			
	Coeff.	*SE*	LLCI	ULCI	Coeff.	*SE*	LLCI	ULCI
T1 Fphubbing	−0.28 ***	0.03	−0.33	−0.23	0.13 ***	0.03	0.08	0.19
T2 RS					−0.21 ***	0.03	−0.26	−0.16
*R* ^2^	0.08				0.08			
*F*	111.44 ***				55.28 ***			

Note: *N* = 1319. Coeff.—coefficient, *SE*—standard error, LLCI—lower limit of the 95% confidence interval, ULCI—upper limit of the 95% confidence interval. The beta values are unstandardized coefficients. Each column is a regression model that predicts the criterion at the top of the column. Fphubbing—father phubbing, RS—relationship satisfaction. *** *p* < 0.001.

**Table 3:** Testing the Moderated Mediation Effect of Father Phubbing on Adolescents’ Depressive Symptoms.

Predictors	Model 1 (T2 RS)				Model 2 (T2 Depressive Symptoms)			
	Coeff.	*SE*	LLCI	ULCI	Coeff.	*SE*	LLCI	ULCI
T1 Fphubbing	−0.26 ***	0.03	−0.32	−0.21	0.13 ***	0.05	0.08	0.19
T1 NTB	−0.04	0.03	−0.09	0.01				
T1 Fphubbing × T1 NTB	−0.08 **	0.03	−0.13	−0.03				
T2 RS					−0.21 ***	0.03	−0.27	−0.16
*R* ^2^	0.09				0.08			
*F*	40.86 ***				56.58 ***			

Note: *N* = 1319. Coeff. = coefficient, *SE* = standard error, LLCI = lower limit of the 95% confidence interval, ULCI = upper limit of the 95% confidence interval. The beta values are standardized coefficients, thus they can be compared to determine the relative strength of different variables in the model. Fphubbing = father phubbing, NTB = need to belong, RS = relationship satisfaction. ** *p* < 0.01. *** *p* < 0.001.

## Data Availability

The raw data supporting the conclusions of this article will be made available by the authors on request.
